# Characterization of presence and activity of microRNAs in the rumen of cattle hints at possible host-microbiota cross-talk mechanism

**DOI:** 10.1038/s41598-022-17445-z

**Published:** 2022-08-15

**Authors:** Sara Ricci, Renée M. Petri, Cátia Pacífico, Ezequias Castillo-Lopez, Raul Rivera-Chacon, Arife Sener-Aydemir, Nicole Reisinger, Qendrim Zebeli, Susanne Kreuzer-Redmer

**Affiliations:** 1 Christian Doppler Laboratory for Innovative Gut Health Concepts of Livestock, Department for Farm Animals and Veterinary Public Health, Institute of Animal Nutrition and Functional Plant Compounds, University of Veterinary Medicine, Vienna, Austria; 2 Agriculture and Agri-Food Canada, Sherbrooke Research and Development Centre, Sherbrooke, QC Canada; 3 Present Address: Biome Diagnostics GmbH, Vienna, Austria; 4 DSM, BIOMIN Research Center, Tulln an der Donau, Austria

**Keywords:** Microbiology, Bacteria, Microbial communities, Gastrointestinal diseases, Extracellular signalling molecules

## Abstract

MicroRNAs (miRNAs), as important post-transcriptional regulators, are ubiquitous in various tissues. The aim of this exploratory study was to determine the presence of miRNAs in rumen fluid, and to investigate the possibility of miRNA-mediated cross-talk within the ruminal ecosystem. Rumen fluid samples from four cannulated Holstein cows were collected during two feeding regimes (forage and high-grain diet) and DNA and RNA were extracted for amplicon and small RNA sequencing. Epithelial biopsies were simultaneously collected to investigate the co-expression of miRNAs in papillae and rumen fluid. We identified 377 miRNAs in rumen fluid and 638 in rumen papillae, of which 373 were shared. Analysis of microbiota revealed 20 genera to be differentially abundant between the two feeding regimes, whereas no difference in miRNAs expression was detected. Correlations with at least one genus were found for 170 miRNAs, of which, 39 were highly significant (r > |0.7| and P < 0.01). Both hierarchical clustering of the correlation matrix and WGCNA analysis identified two main miRNA groups. Putative target and functional prediction analysis for the two groups revealed shared pathways with the predicted metabolic activities of the microbiota. Hence, our study supports the hypothesis of a cross-talk within the rumen at least partly mediated by miRNAs.

## Introduction

The role of microRNAs (miRNAs) as a mechanism of communication between eukaryotic cells and tissues has been the focus of a large body of research in the latest years^1^. These small non-coding RNAs were described for the first time in 1993^2^, and while their main biological function is considered to be the regulation of protein expression in the post-transcriptional phase, as research progresses, we gain more insights on the multiple roles of these molecules in the organism. Their diverse functions include regulation of inflammatory processes and of the gut epithelial structure^3,4^. Furthermore, it has been suggested that the cell-to-cell transfer of miRNAs helps the host to trigger and regulate the immune response^5^. This aspect is of particular importance especially in environments in which microorganisms (including potential pathogens) live in close interaction with the host, such as the gut. Indeed, the gastrointestinal tract (GIT) microbiota is a key element for numerous physiological functions in mammals, influencing complex processes of health and disease. Hence, the cross-talk between the GIT microbiota and its host is essential for the host physiology^6^.

Recently, evidence has supported the hypothesis that miRNAs exert a crucial role in the communication at the interface between the host and the bacteria inhabiting the GIT^7,8^. Several authors have analyzed the relationship between bacteria and the host miRNA, especially in regards to pathogen colonization and the subsequent immune response^9,10^. It is well demonstrated that pathogenic bacteria can interfere with the host miRNAome and regulate the immune response in order to favor their proliferation^11,12^. Although this kind of interaction has been well demonstrated, the inverse, host regulation of microbiota proliferation via miRNAs, has not received the same attention. Liu et al.^13^ showed that the host can shape the microbiota through miRNAs, demonstrating that miRNA deficiency in mice caused major changes in the gut integrity and microbial composition, but also that host miRNAs could enter *Escherichia coli* and *Fusobacterium nucleatum* and regulate bacterial gene transcripts and growth.

The composition and activity of the GIT microbiota is of special importance for ruminants, whose digestive system has evolved in symbiosis with its microbiome to promote the processing and metabolization of nutrients introduced with the diet^14,15^. Given the highly conserved nature of miRNA expression between animals species^16,17^, it is possible that the miRNA based interactions between the host and its microbiota demonstrated in other species exist in cattle as well.

Because of the strong symbiotic relationship between the rumen microbiota and its host, it is conceivable that the cross-talk between the two parts could be externally influenced by changes in the diet. In fact, research assessing the influence of the host on the microbiome has shown that the host regulates ruminal microbiota proliferation and activity even when the microbiome is completely disturbed^18^. Diet is the most influential factor for the rumen ecosystem^14,19^ and it can also influence the expression of circulating miRNAs in cattle^20,21^. When cows are fed a high-grain (HG) diet, the ruminal milieu shifts towards the production of increased quantities of volatile fatty acids (VFAs)^22^ and the host has to adjust to this rapid escalation, with changes in osmolality and pH, as well as alterations in microbe-associated molecular patterns^23^. The host does this by activating diverse defense mechanisms to avoid potential health-related issues associated with low-pH tolerant bacteria^24,25^. Research on miRNAs in cattle has mainly focused on reproduction and production traits, such as muscle, adipose tissue, mammary glands and milk^21,26^. Recent studies have also investigated miRNAs expression in the rumen epithelium, but to the best of our knowledge, miRNAs have never been reported before in the rumen fluid^27–29^.

The aims of this exploratory study were (i) to investigate the presence of miRNAs in the rumen fluid, (ii) to explore the possible effect of a rapid shift from a forage-based to a HG diet on their expression in rumen fluid, (iii) to assess the co-expression of miRNAs between rumen fluid and papillae and finally, (iv) to evaluate the relationship between the microbiota and the expression of miRNAs in the rumen fluid. The detection of interaction patterns between microbial-associated miRNAs, host-associated miRNAs and microbial composition and predicted activity would support our hypothesis that cross-talk between the rumen and its microbiota is at least partly mediated through miRNAs.

## Results

### MicroRNAs

**Figure 1:**
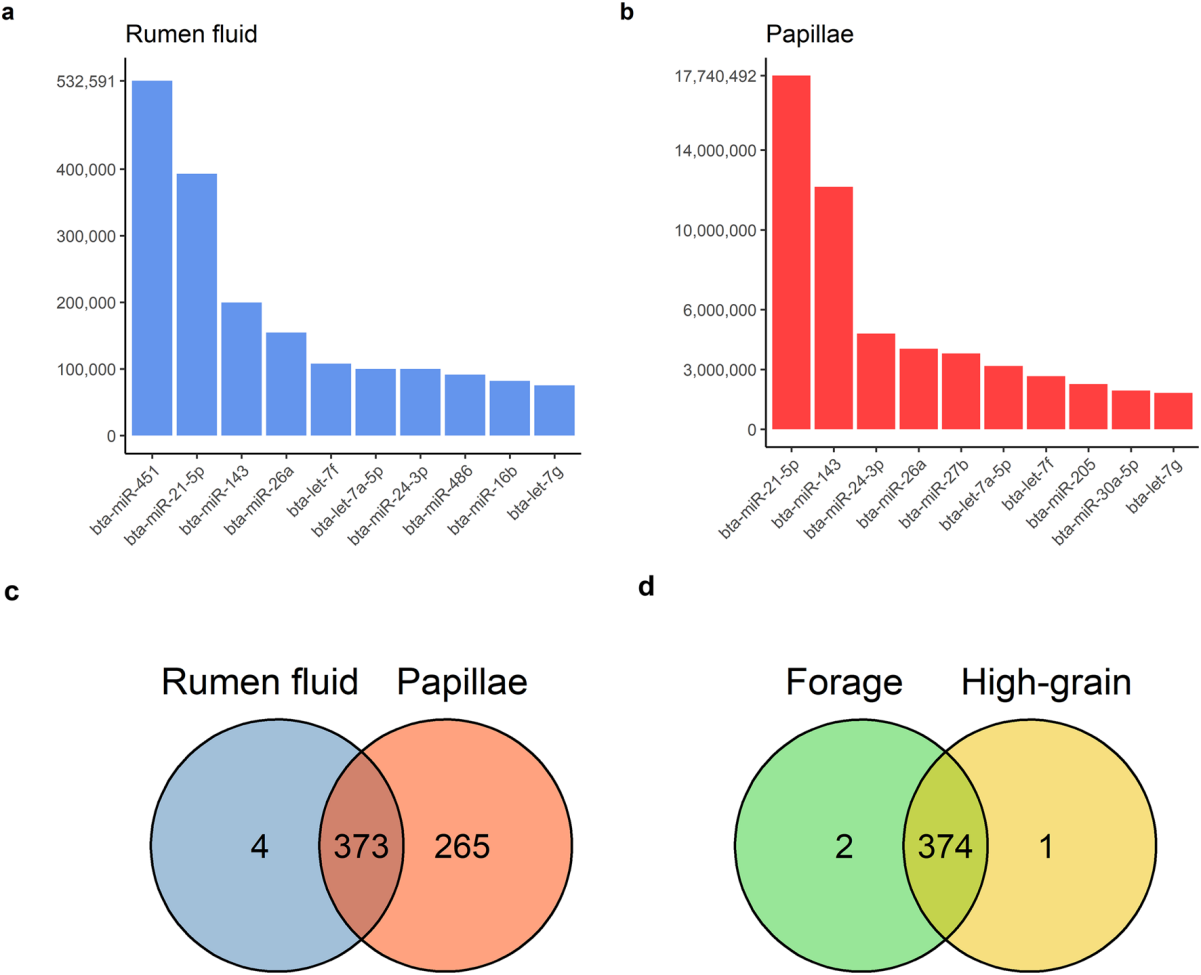
Total read counts of the most expressed miRNAs in the two ruminal niches analyzed (rumen fluid, total RNA input for sequencing: 42–77 ng (**a**); rumen papillae, total RNA input for sequencing: 200 ng per sample (**b**). Venn diagrams showing the number of shared microRNAs between rumen fluid and ruminal papillae (**c**) and shared between forage and high-grain diet in rumen fluid (**d**).

After quality control, 65.03% of the reads for rumen papillae and 45.41% of the reads for rumen fluid samples could be mapped to the bovine genome. A total of 638 miRNAs were identified in the papillae samples and 377 miRNAs were detected in rumen fluid samples (Fig. 1c).

These two ruminal niches shared 373 miRNAs, with four miRNAs being exclusively found in the rumen fluid (bta-miR-137, hsa-miR-137-3p, bta-miR-2285bh and bta-miR-2285ak-5p), albeit expressed in a very limited read count (Fig. 1c). The most expressed miRNAs were overall shared between the two matrices, although with small differences in the top 10 most expressed (Fig. 1a,b). Bta-miR-27b, bta-miR-30a-5p and bta-miR-205 were among the 10 most expressed miRNAs in papillae, but not in rumen fluid. At the same time, the first most abundant miRNA in rumen fluid, bta-miR-451, as well as bta-miR-486 and bta-miR-16b were not among the 10 most expressed in papillae.

The miRNA expression in rumen fluid was consistent during the forage diet and in the high-grain (HG) diet. There were only two miRNAs expressed exclusively in forage diet (bta-miR-2285bh and bta-miR-2285ak-5p) and only one (bta-miR-2285au) expressed solely during HG (Fig. 1d). The same miRNAs were also identified as unique for single animals. Although there were numerical differences in the read count between the two feeding conditions, DESeq2 did not identify any significant differential expression due to the diet in rumen fluid (FDR < 0.05).

### Predicted targets and functional annotation

**Figure 2:**
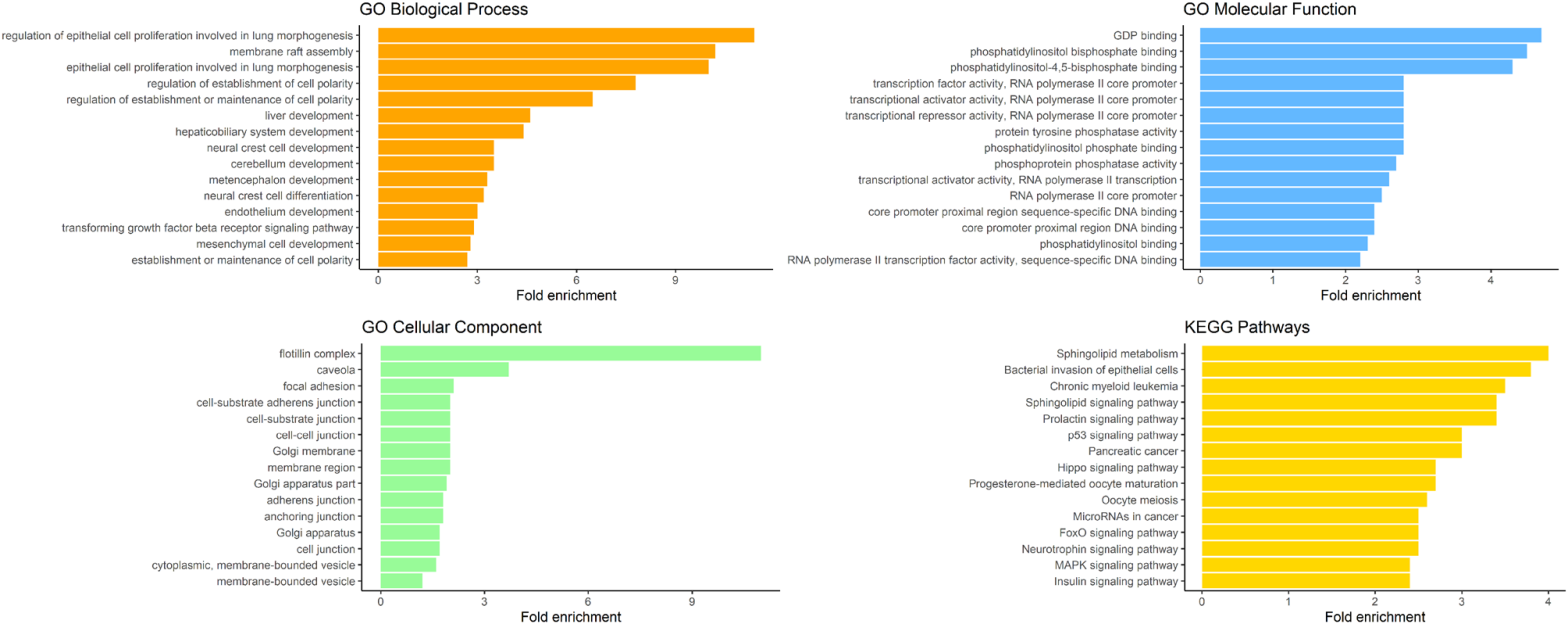
Fold enrichment of the predicted functional annotation of the 10 most positively connected miRNAs for the blue module. Results are presented for Gene Ontology (GO) main biological domains and KEGG pathways. Only the 15 most significantly enriched terms are presented per each category.

TargetScan predicted 749 genes to be targeted by the 10 most expressed miRNAs in rumen fluid. From these, 683 were successfully mapped against the bovine genome. A total of 144 classes for the biological processes (BP) and 11 for the molecular functions (MF) were significantly enriched, while no terms were found to be enriched for the cellular component (CP). A total of 46 Kyoto Encyclopedia of Genes and Genomes (KEGG) pathways were significantly enriched according to the predicted targets for the 10 most expressed miRNAs (Supplementary table T1). The WGCNA analysis assigned the miRNAs to two main modules, identified by colors blue and turquoise (see details in the "MiRNA-microbiota interaction" section). For the blue module, the 10 most positively connected miRNAs had 1051 predicted gene targets (967 mapped to bovine). A total of 248 classes for the BP, 15 terms for the CP and 28 for the MF were found to be enriched. Furthermore, 26 KEGG pathways were significantly enriched (Fig. 2). For the turquoise module, the 10 most positively connected miRNAs had 677 predicted gene targets (589 mapped to bovine), but only 2 classes for the BP were significantly enriched (cellular protein modification process and protein modification process). For the KEGG pathways there was a tendency for the enrichment of FoxO signaling pathway. Four KEGG pathways that were enriched in the blue module (positive related miRNAs) were also identified in the predicted pathways via CowPI (bacterial invasion of epithelial cells, sphingolipid metabolism, insulin signaling pathway, lysosome), although in a very small relative abundance.

### Microbiota

**Figure 3:**
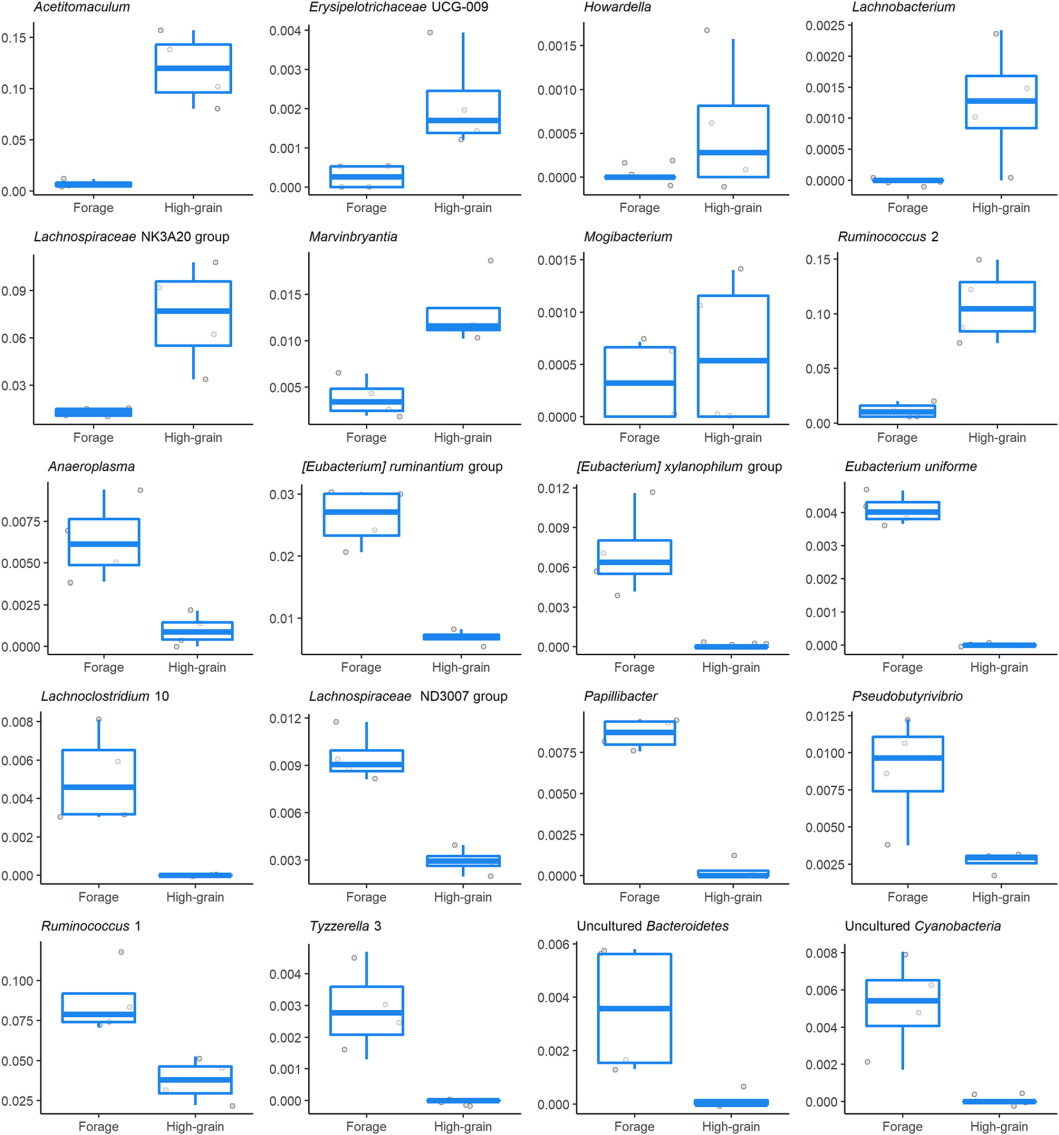
Relative abundances of the 20 genera found to be differentially abundant between the forage and high-grain (HG) feeding in rumen fluid. *Mogibacterium*, *Anaeroplasma*, uncultured *Bacteroidetes*, *Howardella* and *Pseudobutyrivibrio* had P-values between 0.05 and 0.10. For the rest of the genera the P-value was < 0.05. The reported P-values were corrected with the Benjamini–Hochberg method.

**Figure 4:**
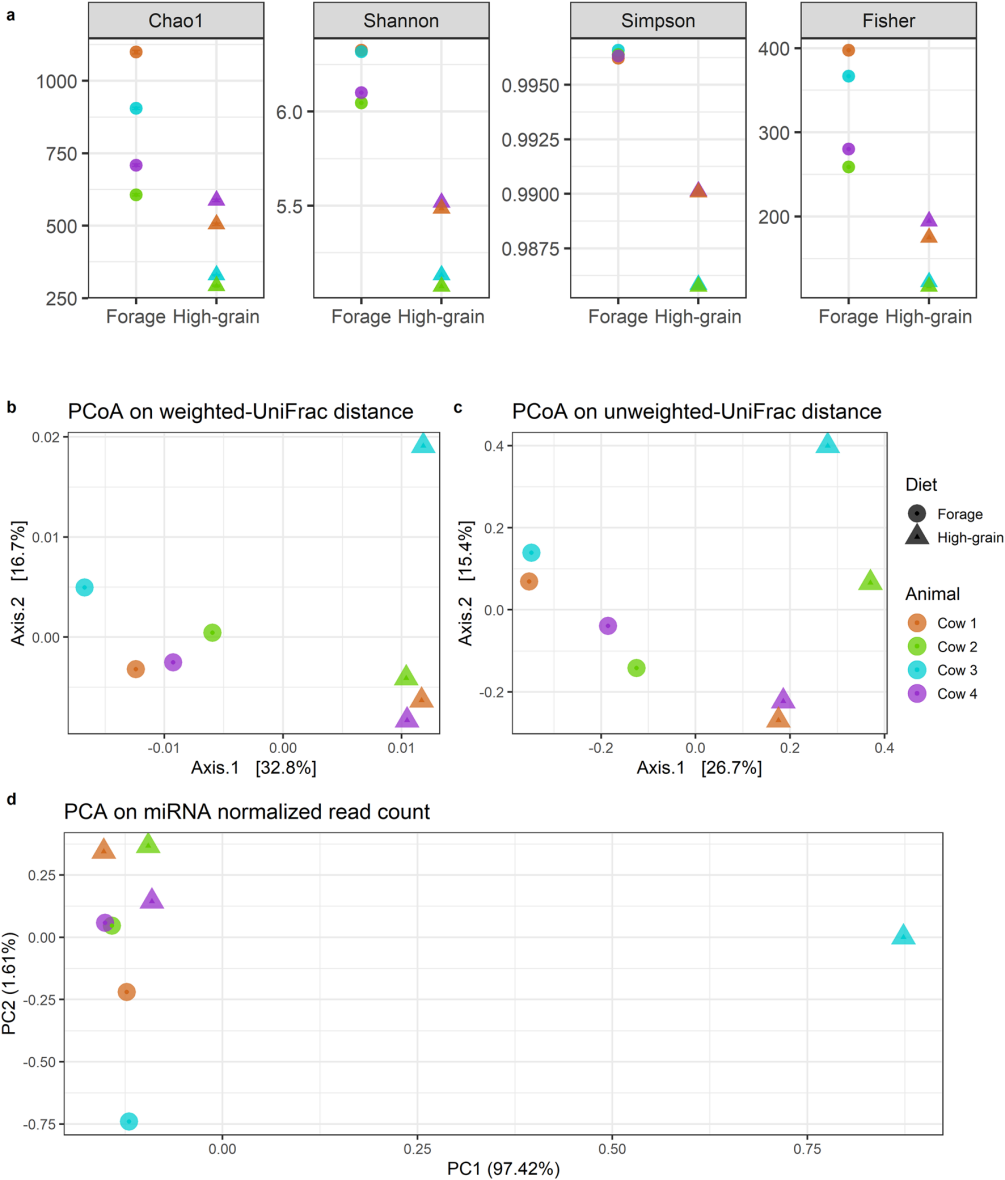
Alpha-diversity indices measured on rumen fluid during forage feeding and high-grain diet (**a**), PCoA on beta-diversity distance matrices (**b**,**c**) and PCA on normalized read counts of the miRNAs identified in rumen fluid (**d**). Chao1 (P = 0.04), Shannon index (P < 0.01), Simpson index (P < 0.01), and Fisher index (P = 0.02) were all reduced in high-grain feeding. Both Weighted UniFrac (P = 0.04) (**b**) and unweighted UniFrac (P = 0.03) (**c**) were influenced by the diet according to ADONIS.

Analyzing the microbiota composition, 2560 amplicon sequence variants (ASVs) were classified in 20 phyla, 73 families and 199 genera. The most abundant phyla were *Firmicutes* (83.9%), *Bacteroidetes* (11.2%) and *Patescibacteria* (2.9%) (Supplementary Fig. S1). *Lachnospiraceae* and *Ruminococcaceae* accounted together for more than 60% abundance at the family level, while the most abundant genera were *Christensenellaceae* R-7 group, *Ruminococcaceae* NK4A214 group, *Prevotella *1, *Acetitomaculum* and *Ruminococcus* 1 and 2. Phylum *Cyanobacteria* and two families (*Bacteroidales* BS11 gut group and uncultured rumen *Gastranaerophilales*) were differentially abundant between the two diets. At the genus level, 20 taxa were found to be differentially abundant from forage to the HG diet, with eight genera significantly increased and 12 reduced (Fig. 3). All alpha-diversity indices tested were affected by the diet change, with a significant reduction of diversity and richness in HG feeding (Fig. 4a). Both weighted and unweighted UniFrac analysis were influenced by the diet according to ADONIS (P = 0.04 and P = 0.03, respectively), while no significant effect due to the individual animal or the interaction between the two parameters was found (Fig. 4b,c). CowPI identified 178 metabolic pathways in the rumen fluid samples, of which only 30 showed a relative abundance above 1%. Some pathways were exclusively detected in one of the two feeding conditions. Inorganic ion transport and metabolism as well as bacterial invasion of epithelial cells were identified only during forage feeding, while carbohydrate digestion and absorption, inositol phosphate metabolism, lipopolysaccharide biosynthesis proteins, amino acid metabolism, glycosphingolipid biosynthesis—globo series and sphingolipid metabolism were only present in HG feeding.

### MiRNA-microbiota interaction

**Figure 5:**
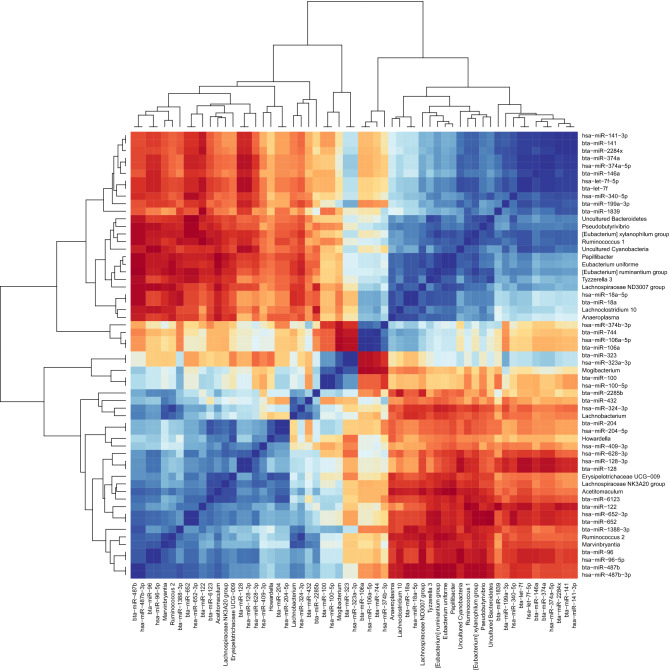
Heatmap of the correlation matrix between 20 differentially abundant genera and a subset of the most significant correlated (P < 0.01 and r > |0.70| for at least one genus) miRNAs identified in the rumen fluid. Taxa unclassified at the genus level were excluded. The hierarchical clustering shows two main clusters. Red corresponds to negative correlations, blue to positive correlations. Darker colors correspond to higher |r| values (range between − 1 and 1).

The miRNAs (normalized read counts) and the differentially abundant genera (relative abundance) per each individual sample were used to compute Pearson correlations. Significant correlations were found for 170 out of 377 identified miRNAs, of which 169 had high correlation scores (r > |0.70|) for at least one genus. Bta-miR-15b had low correlation scores (r < |0.70|) with all taxa. Highly significant correlations (r > |0.7| and P < 0.01) were detected for 39 miRNAs (Fig. 5). Bta-miR-487b (and hsa-miR-487b-3p) showed a strong correlation with the highest number of taxa (15, of which 12 with P < 0.01). Bta-miR-96 (and hsa-miR-96-5p), bta-miR-652 (and hsa-miR-652-3p), bta-miR-18a (and hsa-miR-18a-5p), and bta-miR-6123 showed strong correlation with more than half of the genera. Bta-let-7f and bta-let-7a-5p were among the most expressed miRNAs and were positively correlated with [*Eubacterium*] *xylanophilum* group (r = 0.89, P < 0.01 and R 0.77, P = 0.03, respectively), uncultured *Cyanobacteria* (*Melainabacteria*, order *Gastranaerophilales*, uncultured rumen bacterium) (r = 0.91, P < 0.01 and r = 0.78, P = 0.02, respectively) and *Ruminococcus* 1 (r = 0.90, P < 0.01 and r = 0.75, P = 0.03, respectively). Bta-let-7f was also correlated with *Papillibacter* (r = 0.74, P = 0.04) and with [*Eubacterium*] *ruminantium* group (r = 0.72, P = 0.05). *Ruminococcus 1* (relative abundance 8.7% in forage and 3.8% in HG diet) and *Ruminococcus 2* (relative abundance 1.2% in forage and 10.8% in HG diet) were inversely correlated with the vast majority of miRNAs, including bta-miR-487b and bta-miR-652 (and hsa-miR-652-3p). The hierarchical clustering of the correlation heatmap showed two main clusters, which were also present when reducing the matrix to the most significant correlations (Fig. 5, Supplementary Fig. S2).

**Figure 6:**
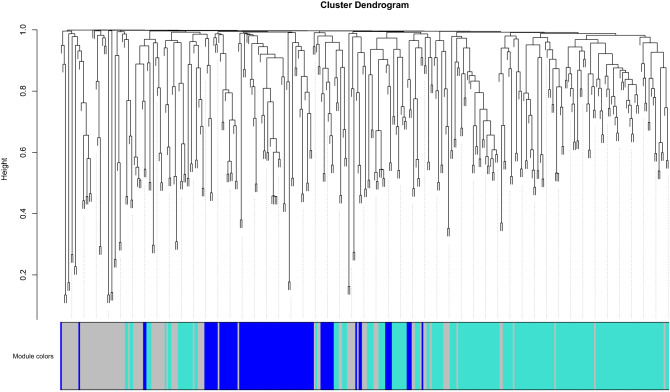
The dendrogram shows the clusters underlying the detection of the two modules identified in the rumen fluid samples. The blue module counted 87 miRNAs, while the turquoise module counted 182 miRNAs. The rest of the miRNAs, that were not significantly connected with either the blue or the turquoise, were grouped into the grey module.

**Figure 7:**
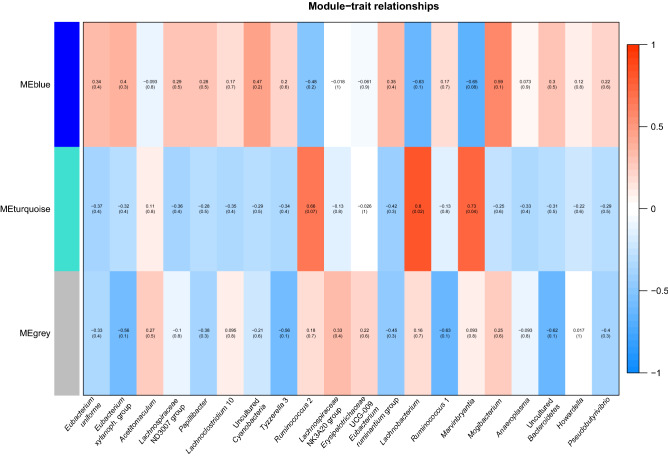
Correlation heatmap showing the relationship between the modules identified by WGCNA and the relative abundance of 20 differentially abundant genera found in rumen fluid (taxa unclassified at the genus level were excluded). Colors represent r values, ranging from − 1 to 1. Darker colors correspond to higher |r| values. The grey module includes miRNAs that were not significantly connected with either the blue or the turquoise module.

Prior to module detection with WGCNA, 10 miRNAs were removed (bta-miR-137, hsa-miR-137-3p, hsa-miR-29a-5p, bta-miR-184, bta-miR-2284y, bta-miR-2285av, bta-miR-11992, bta-miR-2285au, bta-miR-2285bh, bta-miR-2285ak-5p) due to too many missing values in the dataset. The software detected two main modules (identified by colors blue and turquoise), including 87 and 182 miRNAs, respectively (Fig. 6). For 98 miRNAs, no connection was detected (grey module). The most connected miRNAs per each module are shown in Table 1. The majority of genera were inversely correlated with the blue and turquoise modules (Fig. 7). All the taxa that were negatively correlated with the blue module were significantly increased with HG diet. Four genera, *Ruminococcus* 2, *Lachnobacterium*, *Marvinbryantia* and *Mogibacterium*, had the strongest correlations (r > |0.5|) with the modules and were significantly increased with HG diet. The first three were positively correlated with the turquoise module, while the latter was positively correlated with the blue module.

**Table 1:** Most connected miRNAs (positively and negatively) with the two modules identified via WGCNA (turquoise and blue) and their total read counts in rumen fluid samples.

Turquoise	Read count	MM	Blue	Read count	MM
**Positive**					
bta-miR-27b	67,329	0.996	bta-miR-133a	581	0.969
bta-miR-6524	155	0.992	bta-miR-124b	3071	0.966
bta-miR-199a-5p	1651	0.992	bta-miR-124a	3071	0.966
bta-miR-143	199,669	0.992	hsa-miR-145-3p	1653	0.958
bta-miR-7859	125	0.991	bta-miR-9-5p	18,836	0.943
bta-miR-543	136	0.991	bta-miR-16b	81,803	0.926
bta-miR-126-5p	2436	0.990	bta-miR-101	23,298	0.913
bta-miR-152	887	0.990	bta-miR-125a	10,072	0.909
bta-miR-378	14,931	0.990	bta-miR-1	20,374	0.903
bta-miR-205	31,972	0.989	bta-miR-144	10,649	0.903
**Negative**					
bta-miR-486	91,159	− 0.989	bta-miR-450a	117	− 0.929
bta-miR-191	31,352	− 0.985	bta-miR-499	892	− 0.923
bta-miR-93	26,870	− 0.974	bta-miR-6120-3p	376	− 0.920
bta-miR-2285bc	5225	− 0.968	bta-let-7a-3p	687	− 0.912
bta-miR-16a	39,370	− 0.956	bta-miR-193a-3p	285	− 0.911
bta-miR-30b-5p	8439	− 0.954	bta-miR-429	3265	− 0.901
bta-miR-15b	61,988	− 0.949	bta-miR-148a	8198	− 0.888
bta-miR-142-5p	35,176	− 0.932	bta-miR-423-3p	9122	− 0.883
bta-miR-107	2670	− 0.910	bta-miR-181a	5801	− 0.870
bta-miR-150	18,578	− 0.868	hsa-miR-26b-3p	50	− 0.833

MM indicates the module membership: values closer to 1 or − 1 indicate higher connection with the respective module.

The majority (134 out of 170) of the miRNAs that the Pearson correlation found to be significantly correlated with at least one genus were also found belonging to either the blue or the turquoise module. All the miRNAs that were positively associated with the turquoise module were also significant in the Pearson correlation. Four miRNAs (bta-miR-124b, bta-miR-124a, hsa-miR-145-3p and bta-miR-144), which were positively associated with the blue module, were identified also with Pearson correlation. Such miRNAs were negatively correlated with *Ruminococcus* 1 (r = − 0.73 and P = 0.04 for bta-miR-124b, r = − 0.73 and P = 0.04 for bta-miR-124a, r = − 0.71 and P = 0.05 for bta-miR-144) and with uncultured *Cyanobacteria* (r = − 0.73 and P = 0.04 for hsa-miR-145-3p).

Significant correlations were found between 285 miRNAs and at least one microbial predicted pathway. Out of the 39 miRNAs that showed high correlations with the differentially abundant genera, 31 showed significant correlations also with at least one predicted pathway (Supplementary Fig. S3). All the most connected miRNAs identified by WGCNA for both modules showed significant correlations with microbial predicted pathways, except hsa-miR-26b-3p. Bta-miR-361 and bta-miR-7857-3p showed significant correlations with the highest number of pathways (15 and 13, respectively), followed by bta-miR-1307, bta-miR-2284ab, bta-miR-2285by and bta-miR-4286, that showed significant correlations with 12 pathways. Carbon fixation pathways in prokaryotes and lysosome pathway showed correlations with the highest number of miRNAs. Among others, lysosome was positively correlated with bta-miR-2285bc (r = 0.95, P < 0.01) and negatively with bta-miR-142-3p (r = − 0.94, P < 0.01) and bta-miR-29a (r = − 0.99, P < 0.01). Starch and sucrose metabolism pathway was positively correlated with bta-miR-361 (r = 0.93, P < 0.01) and bta-miR-6123 (r = 0.72, P = 0.05). Methane metabolism was correlated with 11 miRNAs, including bta-miR-487b (r = 0.73, P = 0.04) and bta-miR-18a (r = − 0.86, P = 0.01). Negative correlations were found also with bta-miR-2284ab (r = − 0.92, P < 0.01), bta-miR-2285by (r = − 0.92, P < 0.01), bta-miR-760-3p (r = − 0.79, P = 0.02) and bta-miR-655 (r = − 0.77, P = 0.03). Sphingolipid metabolism pathway was positively correlated with bta-miR-100 (r = 0.84, P < 0.01) and bta-miR-424-3p (r = 0.85, P < 0.01). Bta-let-7f and bta-let-7a-5p showed a negative correlation with lysosome (r = − 0.90, P < 0.01 and r = − 0.97, P < 0.01, respectively). In addition to methane metabolism, bta-miR-18a was significantly correlated with ABC transporters (r = − 0.73, P = 0.04), transcription factors (r = − 0.84, P = 0.01), and transporters (r = − 0.76, P = 0.03). Bta-miR-487b was also correlated with ABC transporters (r = 0.81, P = 0.01), amino sugar and nucleotide sugar metabolism (r = 0.72, P = 0.05), transcription factors (r = 0.80, P = 0.02), transporters (0.83, P = 0.01), and peptidoglycan biosynthesis (r = 0.76, P = 0.03).

## Discussion

### Core miRNAome

The presence of miRNAs has been demonstrated in human body fluids and in several organs and tissues in cattle, including the rumen epithelium^30–32^. In the present study, we demonstrated for the first time the presence of miRNAs in rumen fluid of cattle, confirming our hypothesis.

It is, however, still unclear how miRNAs reach extracellular fluids. Literature has often reported the presence of miRNAs in exosomes, which can be isolated in saliva and blood^33,34^. In the case of rumen fluid, a potential mechanism of release could indeed be salivary exosomes: it has in fact been demonstrated that exosomes of bovine origin resist the digestive process in humans^35^. Another possible mechanism could be the release from the rumen epithelium during normal turnover process, in form of apoptotic bodies^1,8^. It is known that HG feeding causes a reshaping of the ruminal epithelium, with an increase in size of the papillae, suggesting an enhanced cellular turnover resulting in a possible increase in cell sloughing and therefore, releasing miRNAs into the rumen lumen^36^. Despite the fact that such process seems to be a mere passive release of small RNAs, our data suggest a more active mechanism. In fact, only a part (373 from 638) of the expressed miRNAs from papillae were present in rumen fluid. Furthermore, also within the shared fraction, the most abundantly expressed miRNAs were still different between the two niches. We also identified four miRNAs to be solely expressed in the rumen fluid, although such findings might be a result of sequencing or bioinformatics errors due to their low read count^37,38^.

The animals that were included in our experiment showed a large individual variation, as indicated by the PCoAs and PCA showing the distribution of the samples for UniFrac distances in microbiota as well as for the normalized miRNA read counts. The individual variation we observed for the miRNAs in the rumen fluid was reflected in the microbial data. One animal in particular tended to have a different miRNA expression profile and microbiota composition in the HG diet, suggesting an important role of the animal specific response to the dietary induced challenge in our study^39^. Nevertheless, our statistical analyses did not confirm any significant difference derived from individual variability. Thus, it seems that the ruminal miRNA expression patterns for the 4 subjects included in our study were overall reasonably conserved, with a consistent response towards the dietary challenge.

We have previously demonstrated that the diet switch from forage to HG triggers a series of reactions at the epithelial level: both transcriptome and miRNAome were altered within a week, suggesting a quick change of metabolism in response to the diet^40^. Two of the miRNAs that were differentially expressed in the ruminal papillae (bta-miR-93 and bta-miR-143) were associated with the turquoise module in the present study. However, surprisingly, our analyses did not find any differentially expressed miRNA in rumen fluid. This is a further indication that the rumen fluid miRNAome is not only a passive reflection of what circulates at the level of the epithelium. Nevertheless, since we only sampled free rumen fluid from the ventral sac of the rumen, it needs to be considered that other fractions of the rumen contents could provide different results. The encouraging findings obtained in our exploratory study warrant further research on the presence of miRNAs in other ruminal compartments and their differential expression in response to the diet.

### Role of the miRNAome in rumen fluid

Assuming that the secretion from the ruminal epithelium towards the lumen is directed, our next aim was to uncover the role of miRNAs in the rumen fluid. Our hypothesis was that there is a cross-talk between the host and its ruminal microbiota, possibly mediated by miRNAs. Previous research has provided evidence of this potential interaction, based on the crucial role of miRNAs found in the early stages of gut development in calves^29,41^. Therefore, we performed network analyses to highlight the possible correlations between the rumen fluid miRNAome and the microbiota composition and predicted pathways. MicroRNAs belonging to the let-7 family were among the most expressed in rumen fluid. Family let-7 is highly conserved among animals and seems to regulate RAS gene expression, which is important for mitosis of the rumen epithelial cells^42–44^. The high abundance of miRNAs belonging to let-7 family in rumen fluid could explain the significant enrichment of the RAS signaling pathway and could indicate an influence on host function. Bta-let-7f and bta-let-7a-5p were identified amongst the most expressed miRNAs in our study as well as in several gut tracts in cattle^27,45^. Interestingly, they were correlated only with a few microbial pathways, but showed, together with bta-miR-18a, an inverse correlation pattern with the microbiota composition compared to the other miRNAs. The relationship of bta-miR-18a with bacteria has been demonstrated both in humans and in cattle, where the expression of bta-miR-18a in the distal jejunum was hypothesized to be involved in *E. coli* shedding^45^. In our study, bta-miR-18a was negatively correlated with four pathways, mostly related to transporters.

Bta-miR-487b was correlated with the highest number of genera in our study and with several predicted pathways, but the relationship of this miRNA with bacteria is not well understood yet. Its expression was hypothesized to be linked to gut microbiota in mice, where miR-487b was found to be less expressed in germ-free mice, but at the same time the presence of lipopolysaccharide (LPS) reduces its expression, thus promoting macrophage-mediated immunity^46,47^. This could explain the negative correlation with Gram-negative taxa such as *Anaeroplasma*, uncultured *Bacteroidales* and *Pseudobutyrivibrio* found in our study. However, bta-miR-487b was also negatively correlated with Gram-positive strains, despite being positively correlated with peptidoglycan biosynthesis pathway. These findings, together with the significant correlations with sugar metabolism, transcription factors and transporters, indicate a potential major role played by bta-miR-487b in the rumen and the further need to explore its possible function in regulating host-microbiota interactions.

Despite few available studies on miRNAs in the gut in cattle, the overall knowledge of the composition of the ruminal miRNAome is still largely unknown. For example, we identified several miRNAs belonging to the miR-2285 family: previous research has confirmed the specificity of members of this family to the rumen, while their function remains unknown^27,49^. Though bta-miR-2285bc was strongly associated with the turquoise module from the WGCNA analysis in our study, it was impossible to determine its target genes through TargetScan. While bta-miR-2285bc showed a correlation only with bacterial lysosome pathway, bta-miR-2285by was correlated with a large number of microbial pathways, indicating a potential important role in regulating microbial activity. Particularly interesting was the negative correlation of bta-miR-2285by with methane metabolism. Together with other negatively correlated miRNAs, bta-miR-2285by could represent a further tool to be evaluated in the challenging research of reducing methane emissions in cattle. The lack of research with a focus on the host-microbiota interaction in the rumen prevents us to further understand and discuss the role of miRNAs belonging to the miR-2285 family despite their strong correlations with the ruminal microbiota. Furthermore, Sommer et al. have found that the microbiota triggers different responses in the different tracts of the gut in mice^48^. Therefore, although miRNAs are very conserved across species, the response that we observed in the rumen might be quite different than the effects elicited in lower tracts of the gut.

### The miRNAome and its relationship with the ruminal microbiota

To get a global view on the interaction of the miRNAome and the ruminal microbiota, we performed canonical hierarchical clustering on the Pearson correlation matrix and WGCNA. Both analyses were in agreement in detecting two main clusters among the samples. While the Pearson correlations allowed us to identify possible connections between single miRNAs and the microbiota abundance, WGCNA analysis was performed with the aim to predict the possible function of such interactions on a broader scale. Therefore, the results relative to the modules need to be interpreted considering the miRNAs as part of a group with potentially similar targets and functions, regardless of their interactions as individual entities. Despite containing less than half the total miRNAs found in rumen fluid, one of the two clusters (blue module) had more target genes and enriched pathways than the other (turquoise module).

A possible indication of cross-talk in the rumen is given by the *Ruminococcus* genus groups 1 and 2 inverse correlations with rumen fluid miRNAs, detected both by WGCNA and Pearson analysis. The sequences formerly classified as *Ruminococcus* have been recently split into the two groups, with the majority (67%) assigned to the first group, including *Ruminococcus flavefaciens* and *Ruminococcus albus*, while the rest was assigned to group 2, in which the core representative is *Ruminococcus bromii*^50^. The main metabolic difference between these groups seems to be the capability to metabolize resistant starch. In fact, while *Ruminococcus bromii* is known for its capability to degrade resistant starch, *Ruminococcus albus* seems to prefer substrates other than amylose^51,52^. Furthermore, bta-miR-361 and bta-miR-6123, which were positively correlated with microbial starch metabolism, showed a negative correlation with *Ruminococcus* 1. Therefore, the diet challenge is probably the main responsible for the differential abundance of *Ruminococcus* 1 and 2 found in our study, while the inverse correlation pattern with miRNAs suggests a possible involvement of the host in this response. Given the symbiotic relationship of *Ruminococcus* with the host, it is essential to understand how the host-microbiota cross-talk could influence their proliferation in the gut, especially in response to dietary challenges^53,54^.

Genera [*Eubacterium*] *xylanophilum* group, uncultured *Cyanobacteria* and *Ruminococcus* 1 were correlated with the highest number of miRNAs, indicating that their presence in the rumen fluid is probably highly regulated by the host. It is interesting that *Cyanobacteria* showed a significant correlation with host-derived miRNAs in this study, combined with the fact that this taxon is usually detected in the rumen, even if in low abundance. This genus was classified as belonging to *Melainabacteria*, a non-photosynthetic class^55^, and although the specific ecological niche and even the precise taxonomy of *Cyanobacteria* are still questioned^56,57^ our findings may indicate an important role played in the rumen by bacteria belonging to this phylum.

### Functional analysis of enriched miRNAs and microbiota

Interestingly, the representative miRNAs of the blue module discovered by the WGCNA analysis had enriched classes associated with the cellular component, while the overall most expressed miRNAs did not seem to exert an effect on such biological domain. Therefore, it seems that the miRNAs that were grouped in the blue module could potentially be responsible for the main metabolic effects, and could be overall more active than the miRNAs belonging to the turquoise module.

One of the most interesting results of our study were the common pathways found enriched in the blue module (deriving from host miRNA expression) and detected also through CowPI (referring to microbiota data). Although the pathways predicted based on 16S microbiota data had very low relative abundances, the fact that the same metabolic processes were significantly enriched due to the activity of the miRNA of the blue module strengthens our hypothesis of a possible cross-talk in the rumen. Two of these pathways (bacterial invasion of epithelial cells and lysosome) evoke the capability of some pathogen bacteria to enter the host cells. Such interaction seems in fact to be regulated by miRNAs: some bacterial strains, such as *Salmonella typhimurium*, *Listeria monocytogenes* and *Mycobacterium* sp., have been demonstrated to mutate miRNA expression in the host cells in order to modify the cellular cytoskeleton and alter the phagocytosis process^58–60^. Both miR-142-3p and miR-29a, that were identified as key components of the cross-talk in the aforementioned studies, were negatively correlated with lysosome bacterial pathway in the present experiment.

Another interesting indication of the possible cross-talk in the rumen was the common finding of sphingolipids associated KEGG pathway both in the microbiota and the host. Although only relatively few bacterial strains, belonging to phyla *Bacteroidetes* and *Proteobacteria*, are capable of synthetizing this particular type of lipids, it seems that sphingolipids can play a role in the host-microbiota interaction. Previous research has in fact demonstrated that bacterial-derived sphingolipids can modulate the host lipid metabolism and sphingolipid de novo biosynthesis, as well as be used as signaling molecules^61,62^. However, the miRNAs that were correlated with sphingolipid metabolism pathway were correlated with taxa belonging to phylum *Firmicutes* in our study, indicating that this interaction mechanism warrants further research in the ruminal environment.

## Conclusion

The results discussed so far depict a fairly suggestive picture of the possible host-microbiota cross-talk mediated by miRNAs. While we recognize the limits of our exploratory study, as well as the lack of research on this topic, we believe that our experiment could serve as basis for further research: experiments with larger sample size and including animals at different stages of lactation could confirm and strengthen our findings. Nonetheless, we believe that the elements gathered in this study support the hypothesis of an existing miRNA-mediated host-microbiota interaction and are strong enough to raise the interest of the scientific community to further investigate this widely unexplored topic^8^. As a matter of fact, some researchers have already delivered evidence on the role of miRNAs in the development of the GIT of calves, recently focusing their interest also on the interaction with the microbiota^32,41,63^. However, studies on adult cattle are still lacking. Our data support the idea that unveiling the mechanisms of cross-talk between the host and the gut microbiota in livestock could provide significant knowledge also for human medicine, as suggested by Malmuthuge and Guan in their recent review work^64^.

In this study, we demonstrated for the first time the presence of miRNAs in rumen fluid, and we propose their potential role as a mechanism of interaction between the host and the ruminal microbiota. We suggest this communication to be bidirectional, with the microbiota influencing the miRNA expression pattern of the host, and the host possibly contributing to shape the gut bacterial profile through the production of specific miRNAs.

## Materials and methods

### Experimental design and animal housing

Four non-lactating ruminally-cannulated Holstein cows (916.3 ± 36.3 kg, 10.0 ± 1.5 years) were used in this study, that was approved by the Institutional Ethics and Animal Welfare Committee of the University of Veterinary Medicine Vienna and the Austrian national authority according to the law for animal experiments (protocol number: BMNWF-68.205/0003-V/3b/2019) and conducted in compliance with ARRIVE guidelines. The cows selected were a subset of a larger group used in a parallel study, in which they served as control animals. The cows were transitioned from a forage diet (baseline) to a high-grain diet (HG) using a step-wise adaptation over a 1-week period. The forage diet consisted of 75% grass silage, 15% corn silage, and 10% grass hay on dry matter (DM) basis. The HG diet included 26.3% grass silage, 8.7% corn silage and 65% concentrate on DM basis. Concentrate mixture consisted of pellets composed of 30.22% barley, 18.1% triticale, 23.08% bakery by-products, 24.0% rapeseed meal, 3.0% molasses, 0.8% mineral-vitamin premix for dairy cattle, 0.5% limestone, and 0. 3% salt. The animals were fed once daily, at 7:00 am. Feed, mineral blocks, and water were available for ad libitum consumption. Cows were housed in a free-stall barn with individual deep cubicles (2.6 × 1.25 m) with straw bedding.

### Sample collection

Samples were collected at baseline, on day 7 of the forage diet, and at HG, on the first day of receiving 65% concentrate in the diet. All samples were collected four hours after the morning feeding. The sampling schedule was aimed to detect fast changes in the miRNA expression in response to the dietary change, as well as to allow the microbiota to maximize its activity after the morning feeding. First, rumen fluid was collected from the ventral sac of the rumen using a 20 ml sterile syringe. Samples were immediately frozen in liquid nitrogen in 2 ml cryotubes. To collect papillae samples, the rumen was manually partially emptied, and the content was stored in insulated plastic bins. An area of rumen wall approximately 20 cm below the fistula was exteriorized through the cannula and washed with PBS (circa 5 × 5 cm). The papillae were cut with sterile scissors close to their basis. The papillae were immediately submerged in PBS and gently shaken for a few seconds. The tissue samples were snap frozen in liquid nitrogen and then placed in 2 ml cryotubes, which were then stored at − 80 °C until further processing. The rumen was refilled after sampling. The equipment was washed and disinfected with ethanol or discarded after every use.

### RNA extraction

Total RNA from rumen fluid samples was extracted using NucleoSpin Plasma miRNA Kit (Macherey–Nagel, Germany) following the manufactures protocol for isolation of small and large RNAs. Minor modifications were introduced to optimize the protocol for rumen fluid: a total of 300 µl were used as starting material, and RNA was eluted with 30 µl of RNase-free water. For papillae samples, RNA was extracted according to the manufacturer’s instructions with minor changes using the NucleoSpin miRNA kit (Macherey–Nagel, Germany), as described by Pacífico et al.^40^. The extraction was performed with 30 mg of papillae starting material, which was disrupted and homogenized in 300 µl lysis buffer with ceramic beads. The RNA was eluted using 50 µl of RNase-free water. All samples were frozen and stored at − 80 °C. Quality control was performed using a chip for small RNAs (Agilent, California, USA) in a Bioanalyzer instrument (Agilent 2100 Bioanalyzer, California, USA). The resulting electropherograms and electronic virtual gel-like images were carefully examined to assess miRNA quantity and quality before sequencing.

### RNA sequencing and bioinformatics

Sequencing was performed using a 50-base single-end approach on a NovaSeq 6000 (Illumina) platform by CeGat GmbH (Tübingen, Germany). Libraries were prepared from 200 ng for papillae samples and from 42 ng up to 76 ng for rumen fluid samples using NETflex Small RNA-Seq Kit (Bioo Scientific). Sequences were demultiplexed with Illumina bcl2fastq (2.20) and adapters were trimmed with Skewer (version 0.2.2). Demultiplexed reads were analyzed using sRNAbench^65,66^, after discarding reads with an average PHRED score below 20 and below 15 nucleotides in length. The software was run in genome mode using the cow genome (UMD3_1_mp)^67^ and miRBase 22 as a reference, to identify bovine (bta) miRNAs and human (hsa) miRNA homologues. Sequences were mapped using Bowtie (v1)^66,68^, allowing two nucleotide mismatches. The resulting files per each sample were filtered based on the read counts using package tidyverse^69^ in R software (R version 4.0.2). The human homologues for which a corresponding bovine miRNA was not identified were included in downstream analyses. The resulting final dataset contained the selected miRNAs on the basis of the minimum read count (10 reads in at least one sample).

TargetScan (release 8.0, September 2021) was used to predict the target genes of the 10 most expressed miRNAs^70^, selecting the target genes with a cumulative weighted context++ score < − 0.4^40^. Database for Annotation, Visualization and Integrated Discovery (DAVID) v6.8 was used to inspect the annotated functions of the predicted target genes using *Bos taurus* as reference genome. Results were inspected for KEGG pathways and Gene ontology (GO). The latter was further investigated in regards to molecular function (MF), biological process (BP), and cellular component (CC). P-values were adjusted with Benjamini–Hochberg method and considered significant if P ≤ 0.05 and trends if 0.05 < P ≤ 0.1.

### Bacterial DNA extraction, sequencing and bioinformatics

For bacterial DNA extraction from rumen fluid, the same protocol as described in Ricci et al.^71^ was used (DNeasy PowerSoil Kit (Qiagen, Germany)) with the same modifications and additional pre-processing steps for mechanical and enzymatic lysis. The concentration of DNA extracted from each sample was measured with Qubit fluorometer (Qubit™ 4 Fluorometer, Thermo Fisher Scientific, USA) using the Qubit™ dsDNA HS Assay Kit (Life Technologies, CA, USA). Targeted 16S rRNA gene sequencing was performed in an external laboratory (Microsynth, Balgach, Switzerland), using the primers 341F-ill (5′-CCTACGGGNGGCWGCAG-3′) and 802R-ill (5′-GACTACHVGGGTATCTAATCC-3′), targeting the V3-V4 hypervariable region of the 16S rRNA gene^72^. Libraries were prepared with 16S Nextera two-step PCR, adding barcodes and Illumina adaptors. Equimolar pools of samples were sequenced using Illumina MiSeq sequencing platform (250 bp paired-end reads). The resulting sequences were demultiplexed, trimmed and merged by Microsynth. Quality of the merged reads was inspected using FastQC^73^ before being analyzed with software QIIME 2 (v. 2020.2)^74^. Quality filtering was performed (minimum PHRED score = 20) before denoising with deblur, trimming the samples at 400 nucleotides^75^. A Naive Bayes classifier was trained for the specific primer sequences to assign taxonomy against the SILVA 132 99% OTU reference database. The sequences were then further filtered to exclude mitochondrial contamination. Alpha- and beta-diversity were calculated and analyzed using phyloseq and vegan packages in R^76,77^. The most representative sequences and the feature table were used for functional prediction of the rumen fluid microbiota through CowPI^78^. Briefly, the software implements the widely known Phylogenetic Investigation of Communities by Reconstruction of Unobserved States (PICRUSt) by combining the datasets of the Global Rumen Census and the Hungate 1000 project, thus targeting the metabolic prediction specifically for ruminal microorganisms^14,79^. The functional prediction was run in the publicly available application in Galaxy (https://share-galaxy.ibers.aber.ac.uk/, v. 1.1.1.0) with the default parameters.

### Network analysis and functional prediction

A subset of bacterial taxa (see “Statistical analyses” section) was used to compute network analysis between miRNAs and microbiota through Weighted gene co-expression network analysis (WGCNA)^80^. The weighted co-expression analysis was run using blockwiseModules function in unsigned clustering mode, with a soft-thresholding power of 14, biweight mid-correlation as correlation method and restricting the proportion of outliers to 0.05. Topological Overlap Measure (TOM) was set as “signed” to account for possible anti-reinforcing connection strengths. The minimum size for module detection was set to 30 and 0.25 was the threshold for dendrogram height cut for module merging. TargetScan and DAVID were used to predict the targets and functions of the main miRNAs belonging to the modules identified by WGCNA. The most important miRNAs per each module were identified based on the Module Membership (MM), which measures how correlated a miRNA is to the module eigengene. If the MM value is close to 1 or -1, it is highly connected to the module. Therefore, the top 10 most connected miRNAs (both positive and negative) were used in TargetScan. The predicted targets with a cumulative weighted context++ score < -0.4 were merged for the 10 most connected miRNAs in both modules and used as input for DAVID. Figures were obtained using R packages ggplot2, ggvenn, and ggpubr^81–83^.

### Statistical analyses

Statistical analyses were performed using R^84^. Samples were examined for most expressed and for shared and unique miRNAs between animals and diets using tidyverse. Differential expression was calculated with DESeq2 package version 1.30.1^85^, using the following formula for the model: Y = cow + diet. Normalization was performed according to the default options, using DESeq2’s median of ratios. Counts were divided by sample-specific size factors determined by median ratio of miRNA counts relative to geometric mean per miRNA. Alpha-diversity indices were analyzed with lmer function of lme4 package, with diet as fixed effect and cow as random effect^86^. Beta-diversity was tested for the effect of diet, cow and their interaction using ADONIS function from vegan package. Microbiome Multivariable Associations with Linear Models (MaAsLin2 version 0.04) package in R^87^ was used to detect differentially abundant taxa from forage to HG diet. The model was run using Centered Log-Ratio (CLR) normalization and Linear Model (LM) method, with diet as fixed effect and individual animal as random effect. P-values were corrected with Benjamini–Hochberg method^88^, and were considered significant if Q ≤ 0.1. The resulting differentially abundant genera were used to compute Pearson correlations with expression of miRNAs (normalized read counts), using rcorr function of R package Hmisc^89^. Strong correlations were defined as r > |0.7| and P < 0.01. The same subset of bacterial taxa, excluding the unclassified genera, was used to compute network analysis through (WGCNA). Pearson correlations were also performed for the miRNA normalized read counts and the relative abundances of the 30 most abundant microbial predicted pathways and of the 4 pathways that were found in common between the miRNAs functional prediction and CowPI predictions.

## Supplementary Information

Supplementary Information.

## Data Availability

Sequencing data are available at NCBI Sequence Read Archive (SRA) and Gene Expression Omnibus (GEO) databases with accession numbers PRJNA802085 for the 16S rRNA gene and at GSE195757 for the rumen fluid miRNAs.
