# Supplementary material for: Characterization of presence and activity of microRNAs in the rumen of cattle hints at possible host-microbiota cross-talk mechanism
Source: Sci Rep. 2022 Aug 15;12:13812. doi: 10.1038/s41598-022-17445-z (PMC9378797; doi:10.1038/s41598-022-17445-z)
Supplement: Supplementary file 1 — Supplementary Information. [file 41598_2022_17445_MOESM1_ESM.docx]

**Supplementary material**

**Supplementary Table T1.** Enriched GO classes and KEGG pathways resulting from the target and functional prediction for the 10 most expressed miRNAs in the rumen fluid. (Count = number of genes involved per each term; % = percentage of genes involved per each term; q-value = p-value adjusted with Benjamini and Hochberg method).

| **Category** | **Term** | **Count** | **%** | **Fold Enrichment** | **q-value** |
| --- | --- | --- | --- | --- | --- |
| KEGG_PATHWAY | Thyroid cancer | 8 | 1.2 | 7.3 | < 0.01 |
| KEGG_PATHWAY | Dorso-ventral axis formation | 6 | 0.9 | 6.6 | 0.01 |
| KEGG_PATHWAY | Acute myeloid leukemia | 12 | 1.8 | 6.1 | < 0.01 |
| KEGG_PATHWAY | Bladder cancer | 8 | 1.2 | 5.7 | < 0.01 |
| KEGG_PATHWAY | FoxO signaling pathway | 26 | 3.8 | 5.6 | < 0.01 |
| KEGG_PATHWAY | Prostate cancer | 17 | 2.5 | 5.6 | < 0.01 |
| KEGG_PATHWAY | Endometrial cancer | 10 | 1.5 | 5.6 | < 0.01 |
| KEGG_PATHWAY | Melanoma | 14 | 2 | 5.5 | < 0.01 |
| KEGG_PATHWAY | Glioma | 12 | 1.8 | 5.2 | < 0.01 |
| KEGG_PATHWAY | Progesterone-mediated oocyte maturation | 15 | 2.2 | 4.8 | < 0.01 |
| KEGG_PATHWAY | p53 signaling pathway | 12 | 1.8 | 4.8 | < 0.01 |
| KEGG_PATHWAY | Colorectal cancer | 11 | 1.6 | 4.7 | < 0.01 |
| KEGG_PATHWAY | Pancreatic cancer | 10 | 1.5 | 4.4 | < 0.01 |
| KEGG_PATHWAY | Chronic myeloid leukemia | 11 | 1.6 | 4.3 | < 0.01 |
| KEGG_PATHWAY | Neurotrophin signaling pathway | 18 | 2.6 | 4.1 | < 0.01 |
| KEGG_PATHWAY | Basal cell carcinoma | 8 | 1.2 | 4.1 | 0.02 |
| KEGG_PATHWAY | MicroRNAs in cancer | 35 | 5.1 | 4 | < 0.01 |
| KEGG_PATHWAY | Melanogenesis | 14 | 2 | 4 | < 0.01 |
| KEGG_PATHWAY | Signaling pathways regulating pluripotency of stem cells | 19 | 2.8 | 3.9 | < 0.01 |
| KEGG_PATHWAY | Long-term potentiation | 9 | 1.3 | 3.9 | 0.01 |
| KEGG_PATHWAY | TGF-beta signaling pathway | 11 | 1.6 | 3.8 | 0.01 |
| KEGG_PATHWAY | Apoptosis | 8 | 1.2 | 3.7 | 0.03 |
| KEGG_PATHWAY | Central carbon metabolism in cancer | 8 | 1.2 | 3.7 | 0.03 |
| KEGG_PATHWAY | Oocyte meiosis | 14 | 2 | 3.5 | < 0.01 |
| KEGG_PATHWAY | Prolactin signaling pathway | 9 | 1.3 | 3.5 | 0.03 |
| KEGG_PATHWAY | Renal cell carcinoma | 8 | 1.2 | 3.4 | 0.04 |
| KEGG_PATHWAY | Hepatitis B | 17 | 2.5 | 3.3 | < 0.01 |
| KEGG_PATHWAY | HIF-1 signaling pathway | 11 | 1.6 | 3.3 | 0.01 |
| KEGG_PATHWAY | Cell cycle | 14 | 2 | 3.2 | < 0.01 |
| KEGG_PATHWAY | MAPK signaling pathway | 28 | 4.1 | 3.1 | < 0.01 |
| KEGG_PATHWAY | Proteoglycans in cancer | 22 | 3.2 | 3.1 | < 0.01 |
| KEGG_PATHWAY | PI3K-Akt signaling pathway | 37 | 5.4 | 3 | < 0.01 |
| KEGG_PATHWAY | Hippo signaling pathway | 16 | 2.3 | 3 | < 0.01 |
| KEGG_PATHWAY | Wnt signaling pathway | 14 | 2 | 2.9 | 0.01 |
| KEGG_PATHWAY | Sphingolipid signaling pathway | 12 | 1.8 | 2.8 | 0.02 |
| KEGG_PATHWAY | Thyroid hormone signaling pathway | 11 | 1.6 | 2.8 | 0.03 |
| KEGG_PATHWAY | Pathways in cancer | 38 | 5.6 | 2.7 | < 0.01 |
| KEGG_PATHWAY | Transcriptional misregulation in cancer | 16 | 2.3 | 2.7 | 0.01 |
| KEGG_PATHWAY | Insulin signaling pathway | 13 | 1.9 | 2.7 | 0.02 |
| KEGG_PATHWAY | Ras signaling pathway | 21 | 3.1 | 2.5 | < 0.01 |
| KEGG_PATHWAY | Focal adhesion | 18 | 2.6 | 2.5 | 0.01 |
| KEGG_PATHWAY | Rap1 signaling pathway | 18 | 2.6 | 2.4 | 0.01 |
| KEGG_PATHWAY | Measles | 12 | 1.8 | 2.4 | 0.05 |
| KEGG_PATHWAY | cAMP signaling pathway | 15 | 2.2 | 2.2 | 0.05 |
| KEGG_PATHWAY | HTLV-I infection | 20 | 2.9 | 2.1 | 0.02 |
| KEGG_PATHWAY | Cytokine-cytokine receptor interaction | 16 | 2.3 | 2.1 | 0.05 |
| GOTERM_BP_FAT | activin receptor signaling pathway | 8 | 1.2 | 7.5 | < 0.01 |
| GOTERM_BP_FAT | epithelial tube branching involved in lung morphogenesis | 7 | 1 | 7.1 | 0.02 |
| GOTERM_BP_FAT | forelimb morphogenesis | 8 | 1.2 | 6.2 | 0.01 |
| GOTERM_BP_FAT | lung morphogenesis | 11 | 1.6 | 5.9 | < 0.01 |
| GOTERM_BP_FAT | G1/S transition of mitotic cell cycle | 17 | 2.5 | 4.4 | < 0.01 |
| GOTERM_BP_FAT | cell cycle G1/S phase transition | 18 | 2.6 | 4 | < 0.01 |
| GOTERM_BP_FAT | negative regulation of translation | 14 | 2 | 3.7 | 0.01 |
| GOTERM_BP_FAT | cell cycle arrest | 13 | 1.9 | 3.6 | 0.01 |
| GOTERM_BP_FAT | negative regulation of cellular amide metabolic process | 14 | 2 | 3.5 | 0.01 |
| GOTERM_BP_FAT | cartilage development | 17 | 2.5 | 3.1 | 0.01 |
| GOTERM_BP_FAT | cellular response to transforming growth factor beta stimulus | 16 | 2.3 | 3 | 0.01 |
| GOTERM_BP_FAT | response to transforming growth factor beta | 16 | 2.3 | 3 | 0.02 |
| GOTERM_BP_FAT | mitotic cell cycle phase transition | 25 | 3.7 | 2.9 | < 0.01 |
| GOTERM_BP_FAT | morphogenesis of a branching epithelium | 17 | 2.5 | 2.8 | 0.02 |
| GOTERM_BP_FAT | branching morphogenesis of an epithelial tube | 14 | 2 | 2.8 | 0.05 |
| GOTERM_BP_FAT | cell cycle phase transition | 26 | 3.8 | 2.7 | < 0.01 |
| GOTERM_BP_FAT | transmembrane receptor protein serine/threonine kinase signaling pathway | 25 | 3.7 | 2.7 | < 0.01 |
| GOTERM_BP_FAT | respiratory system development | 17 | 2.5 | 2.7 | 0.03 |
| GOTERM_BP_FAT | protein polyubiquitination | 15 | 2.2 | 2.7 | 0.05 |
| GOTERM_BP_FAT | positive regulation of cellular catabolic process | 15 | 2.2 | 2.7 | 0.05 |
| GOTERM_BP_FAT | lung development | 15 | 2.2 | 2.7 | 0.05 |
| GOTERM_BP_FAT | respiratory tube development | 15 | 2.2 | 2.7 | 0.05 |
| GOTERM_BP_FAT | connective tissue development | 18 | 2.6 | 2.6 | 0.02 |
| GOTERM_BP_FAT | morphogenesis of a branching structure | 17 | 2.5 | 2.6 | 0.04 |
| GOTERM_BP_FAT | fat cell differentiation | 16 | 2.3 | 2.6 | 0.05 |
| GOTERM_BP_FAT | regulation of protein serine/threonine kinase activity | 26 | 3.8 | 2.4 | 0.01 |
| GOTERM_BP_FAT | regulation of cellular catabolic process | 21 | 3.1 | 2.4 | 0.03 |
| GOTERM_BP_FAT | cellular response to growth factor stimulus | 34 | 5 | 2.3 | < 0.01 |
| GOTERM_BP_FAT | cell division | 28 | 4.1 | 2.3 | 0.01 |
| GOTERM_BP_FAT | tube morphogenesis | 26 | 3.8 | 2.3 | 0.01 |
| GOTERM_BP_FAT | positive regulation of cell cycle | 20 | 2.9 | 2.3 | 0.04 |
| GOTERM_BP_FAT | enzyme linked receptor protein signaling pathway | 49 | 7.2 | 2.2 | < 0.01 |
| GOTERM_BP_FAT | developmental growth | 38 | 5.6 | 2.2 | < 0.01 |
| GOTERM_BP_FAT | response to growth factor | 34 | 5 | 2.2 | < 0.01 |
| GOTERM_BP_FAT | cell cycle process | 65 | 9.5 | 2.1 | < 0.01 |
| GOTERM_BP_FAT | mitotic cell cycle | 45 | 6.6 | 2.1 | < 0.01 |
| GOTERM_BP_FAT | mitotic cell cycle process | 40 | 5.9 | 2.1 | < 0.01 |
| GOTERM_BP_FAT | regulation of kinase activity | 41 | 6 | 2.1 | < 0.01 |
| GOTERM_BP_FAT | regulation of protein kinase activity | 38 | 5.6 | 2.1 | < 0.01 |
| GOTERM_BP_FAT | tube development | 38 | 5.6 | 2.1 | < 0.01 |
| GOTERM_BP_FAT | positive regulation of kinase activity | 25 | 3.7 | 2.1 | 0.03 |
| GOTERM_BP_FAT | positive regulation of locomotion | 25 | 3.7 | 2.1 | 0.04 |
| GOTERM_BP_FAT | cell cycle | 77 | 11.3 | 2 | < 0.01 |
| GOTERM_BP_FAT | growth | 52 | 7.6 | 2 | < 0.01 |
| GOTERM_BP_FAT | negative regulation of protein metabolic process | 53 | 7.8 | 2 | < 0.01 |
| GOTERM_BP_FAT | regulation of transferase activity | 45 | 6.6 | 2 | < 0.01 |
| GOTERM_BP_FAT | positive regulation of cell proliferation | 43 | 6.3 | 2 | < 0.01 |
| GOTERM_BP_FAT | negative regulation of phosphorus metabolic process | 30 | 4.4 | 2 | 0.03 |
| GOTERM_BP_FAT | negative regulation of phosphate metabolic process | 30 | 4.4 | 2 | 0.03 |
| GOTERM_BP_FAT | regulation of catabolic process | 27 | 4 | 2 | 0.03 |
| GOTERM_BP_FAT | regulation of cell cycle process | 29 | 4.2 | 2 | 0.03 |
| GOTERM_BP_FAT | skeletal system development | 28 | 4.1 | 2 | 0.04 |
| GOTERM_BP_FAT | regulation of cell cycle | 47 | 6.9 | 1.9 | < 0.01 |
| GOTERM_BP_FAT | negative regulation of cellular protein metabolic process | 48 | 7 | 1.9 | < 0.01 |
| GOTERM_BP_FAT | organ morphogenesis | 52 | 7.6 | 1.9 | < 0.01 |
| GOTERM_BP_FAT | regulation of growth | 31 | 4.5 | 1.9 | 0.05 |
| GOTERM_BP_FAT | negative regulation of gene expression | 66 | 9.7 | 1.8 | < 0.01 |
| GOTERM_BP_FAT | negative regulation of cellular macromolecule biosynthetic process | 60 | 8.8 | 1.8 | < 0.01 |
| GOTERM_BP_FAT | positive regulation of signal transduction | 66 | 9.7 | 1.8 | < 0.01 |
| GOTERM_BP_FAT | negative regulation of macromolecule biosynthetic process | 62 | 9.1 | 1.8 | < 0.01 |
| GOTERM_BP_FAT | epithelium development | 54 | 7.9 | 1.8 | < 0.01 |
| GOTERM_BP_FAT | positive regulation of intracellular signal transduction | 44 | 6.4 | 1.8 | 0.02 |
| GOTERM_BP_FAT | protein ubiquitination | 35 | 5.1 | 1.8 | 0.04 |
| GOTERM_BP_FAT | vasculature development | 33 | 4.8 | 1.8 | 0.04 |
| GOTERM_BP_FAT | regulation of MAPK cascade | 33 | 4.8 | 1.8 | 0.05 |
| GOTERM_BP_FAT | regulation of protein metabolic process | 119 | 17.4 | 1.7 | < 0.01 |
| GOTERM_BP_FAT | regulation of cellular protein metabolic process | 112 | 16.4 | 1.7 | < 0.01 |
| GOTERM_BP_FAT | negative regulation of macromolecule metabolic process | 97 | 14.2 | 1.7 | < 0.01 |
| GOTERM_BP_FAT | protein phosphorylation | 82 | 12 | 1.7 | < 0.01 |
| GOTERM_BP_FAT | negative regulation of cellular biosynthetic process | 63 | 9.2 | 1.7 | < 0.01 |
| GOTERM_BP_FAT | regulation of protein phosphorylation | 64 | 9.4 | 1.7 | < 0.01 |
| GOTERM_BP_FAT | negative regulation of biosynthetic process | 63 | 9.2 | 1.7 | < 0.01 |
| GOTERM_BP_FAT | regulation of cell proliferation | 67 | 9.8 | 1.7 | < 0.01 |
| GOTERM_BP_FAT | positive regulation of signaling | 68 | 10 | 1.7 | < 0.01 |
| GOTERM_BP_FAT | negative regulation of nitrogen compound metabolic process | 63 | 9.2 | 1.7 | < 0.01 |
| GOTERM_BP_FAT | regulation of phosphorylation | 67 | 9.8 | 1.7 | < 0.01 |
| GOTERM_BP_FAT | positive regulation of cell communication | 67 | 9.8 | 1.7 | < 0.01 |
| GOTERM_BP_FAT | peptidyl-amino acid modification | 51 | 7.5 | 1.7 | 0.01 |
| GOTERM_BP_FAT | regulation of anatomical structure morphogenesis | 48 | 7 | 1.7 | 0.02 |
| GOTERM_BP_FAT | positive regulation of developmental process | 51 | 7.5 | 1.7 | 0.02 |
| GOTERM_BP_FAT | protein modification process | 160 | 23.4 | 1.6 | < 0.01 |
| GOTERM_BP_FAT | cellular protein modification process | 160 | 23.4 | 1.6 | < 0.01 |
| GOTERM_BP_FAT | negative regulation of metabolic process | 103 | 15.1 | 1.6 | < 0.01 |
| GOTERM_BP_FAT | cell surface receptor signaling pathway | 106 | 15.5 | 1.6 | < 0.01 |
| GOTERM_BP_FAT | negative regulation of cellular metabolic process | 96 | 14.1 | 1.6 | < 0.01 |
| GOTERM_BP_FAT | regulation of protein modification process | 77 | 11.3 | 1.6 | < 0.01 |
| GOTERM_BP_FAT | cell proliferation | 77 | 11.3 | 1.6 | < 0.01 |
| GOTERM_BP_FAT | phosphorylation | 88 | 12.9 | 1.6 | < 0.01 |
| GOTERM_BP_FAT | regulation of phosphate metabolic process | 76 | 11.1 | 1.6 | < 0.01 |
| GOTERM_BP_FAT | regulation of phosphorus metabolic process | 76 | 11.1 | 1.6 | < 0.01 |
| GOTERM_BP_FAT | nervous system development | 87 | 12.7 | 1.6 | < 0.01 |
| GOTERM_BP_FAT | regulation of catalytic activity | 79 | 11.6 | 1.6 | < 0.01 |
| GOTERM_BP_FAT | regulation of transcription from RNA polymerase II promoter | 74 | 10.8 | 1.6 | < 0.01 |
| GOTERM_BP_FAT | cellular response to organic substance | 79 | 11.6 | 1.6 | < 0.01 |
| GOTERM_BP_FAT | positive regulation of protein metabolic process | 67 | 9.8 | 1.6 | 0.01 |
| GOTERM_BP_FAT | positive regulation of cellular protein metabolic process | 63 | 9.2 | 1.6 | 0.01 |
| GOTERM_BP_FAT | neurogenesis | 64 | 9.4 | 1.6 | 0.01 |
| GOTERM_BP_FAT | regulation of programmed cell death | 59 | 8.6 | 1.6 | 0.02 |
| GOTERM_BP_FAT | generation of neurons | 59 | 8.6 | 1.6 | 0.02 |
| GOTERM_BP_FAT | cell-cell signaling | 54 | 7.9 | 1.6 | 0.02 |
| GOTERM_BP_FAT | regulation of apoptotic process | 58 | 8.5 | 1.6 | 0.03 |
| GOTERM_BP_FAT | positive regulation of protein modification process | 50 | 7.3 | 1.6 | 0.04 |
| GOTERM_BP_FAT | positive regulation of phosphorus metabolic process | 48 | 7 | 1.6 | 0.04 |
| GOTERM_BP_FAT | positive regulation of phosphate metabolic process | 48 | 7 | 1.6 | 0.04 |
| GOTERM_BP_FAT | cellular response to endogenous stimulus | 46 | 6.7 | 1.6 | 0.04 |
| GOTERM_BP_FAT | anatomical structure formation involved in morphogenesis | 54 | 7.9 | 1.6 | 0.04 |
| GOTERM_BP_FAT | neuron differentiation | 53 | 7.8 | 1.6 | 0.04 |
| GOTERM_BP_FAT | regulation of gene expression | 157 | 23 | 1.5 | < 0.01 |
| GOTERM_BP_FAT | regulation of nitrogen compound metabolic process | 155 | 22.7 | 1.5 | < 0.01 |
| GOTERM_BP_FAT | regulation of cellular macromolecule biosynthetic process | 143 | 20.9 | 1.5 | < 0.01 |
| GOTERM_BP_FAT | regulation of macromolecule biosynthetic process | 145 | 21.2 | 1.5 | < 0.01 |
| GOTERM_BP_FAT | regulation of signal transduction | 113 | 16.5 | 1.5 | < 0.01 |
| GOTERM_BP_FAT | regulation of cell communication | 119 | 17.4 | 1.5 | < 0.01 |
| GOTERM_BP_FAT | regulation of signaling | 120 | 17.6 | 1.5 | < 0.01 |
| GOTERM_BP_FAT | transcription. DNA-templated | 118 | 17.3 | 1.5 | < 0.01 |
| GOTERM_BP_FAT | regulation of RNA biosynthetic process | 125 | 18.3 | 1.5 | < 0.01 |
| GOTERM_BP_FAT | phosphorus metabolic process | 118 | 17.3 | 1.5 | < 0.01 |
| GOTERM_BP_FAT | phosphate-containing compound metabolic process | 117 | 17.1 | 1.5 | < 0.01 |
| GOTERM_BP_FAT | positive regulation of macromolecule metabolic process | 111 | 16.3 | 1.5 | < 0.01 |
| GOTERM_BP_FAT | regulation of molecular function | 98 | 14.3 | 1.5 | < 0.01 |
| GOTERM_BP_FAT | intracellular signal transduction | 105 | 15.4 | 1.5 | < 0.01 |
| GOTERM_BP_FAT | regulation of cell differentiation | 67 | 9.8 | 1.5 | 0.02 |
| GOTERM_BP_FAT | cell development | 83 | 12.2 | 1.5 | 0.02 |
| GOTERM_BP_FAT | transcription from RNA polymerase II promoter | 66 | 9.7 | 1.5 | 0.02 |
| GOTERM_BP_FAT | positive regulation of response to stimulus | 75 | 11 | 1.5 | 0.02 |
| GOTERM_BP_FAT | apoptotic process | 65 | 9.5 | 1.5 | 0.03 |
| GOTERM_BP_FAT | regulation of multicellular organismal development | 71 | 10.4 | 1.5 | 0.03 |
| GOTERM_BP_FAT | regulation of intracellular signal transduction | 68 | 10 | 1.5 | 0.04 |
| GOTERM_BP_FAT | cellular macromolecule biosynthetic process | 185 | 27.1 | 1.4 | < 0.01 |
| GOTERM_BP_FAT | regulation of nucleobase-containing compound metabolic process | 141 | 20.6 | 1.4 | < 0.01 |
| GOTERM_BP_FAT | regulation of RNA metabolic process | 129 | 18.9 | 1.4 | < 0.01 |
| GOTERM_BP_FAT | regulation of nucleic acid-templated transcription | 124 | 18.2 | 1.4 | < 0.01 |
| GOTERM_BP_FAT | RNA biosynthetic process | 131 | 19.2 | 1.4 | < 0.01 |
| GOTERM_BP_FAT | regulation of transcription. DNA-templated | 123 | 18 | 1.4 | < 0.01 |
| GOTERM_BP_FAT | nucleic acid-templated transcription | 129 | 18.9 | 1.4 | < 0.01 |
| GOTERM_BP_FAT | positive regulation of metabolic process | 116 | 17 | 1.4 | < 0.01 |
| GOTERM_BP_FAT | nucleobase-containing compound biosynthetic process | 142 | 20.8 | 1.4 | < 0.01 |
| GOTERM_BP_FAT | heterocycle biosynthetic process | 144 | 21.1 | 1.4 | < 0.01 |
| GOTERM_BP_FAT | aromatic compound biosynthetic process | 144 | 21.1 | 1.4 | < 0.01 |
| GOTERM_BP_FAT | positive regulation of cellular metabolic process | 107 | 15.7 | 1.4 | 0.01 |
| GOTERM_BP_FAT | cellular response to chemical stimulus | 90 | 13.2 | 1.4 | 0.02 |
| GOTERM_BP_FAT | response to organic substance | 88 | 12.9 | 1.4 | 0.05 |
| GOTERM_BP_FAT | gene expression | 181 | 26.5 | 1.3 | < 0.01 |
| GOTERM_BP_FAT | RNA metabolic process | 153 | 22.4 | 1.3 | 0.01 |
| GOTERM_MF_FAT | translation repressor activity. nucleic acid binding | 4 | 0.6 | 20.5 | 0.05 |
| GOTERM_MF_FAT | translation regulator activity. nucleic acid binding | 5 | 0.7 | 14.2 | 0.02 |
| GOTERM_MF_FAT | translation repressor activity | 6 | 0.9 | 11 | 0.01 |
| GOTERM_MF_FAT | translation regulator activity | 8 | 1.2 | 9.3 | < 0.01 |
| GOTERM_MF_FAT | protein serine/threonine kinase activity | 34 | 5 | 2.4 | < 0.01 |
| GOTERM_MF_FAT | protein kinase activity | 45 | 6.6 | 2.1 | < 0.01 |
| GOTERM_MF_FAT | RNA polymerase II transcription factor activity. sequence-specific DNA binding | 42 | 6.1 | 2 | < 0.01 |
| GOTERM_MF_FAT | phosphotransferase activity. alcohol group as acceptor | 47 | 6.9 | 1.9 | < 0.01 |
| GOTERM_MF_FAT | kinase activity | 51 | 7.5 | 1.8 | < 0.01 |
| GOTERM_MF_FAT | transcription factor activity. sequence-specific DNA binding | 60 | 8.8 | 1.7 | < 0.01 |
| GOTERM_MF_FAT | nucleic acid binding transcription factor activity | 60 | 8.8 | 1.7 | < 0.01 |

**Supplementary Figure S1.** Relative abundance of the 10 most abundant phyla per each animal at the two feeding conditions.


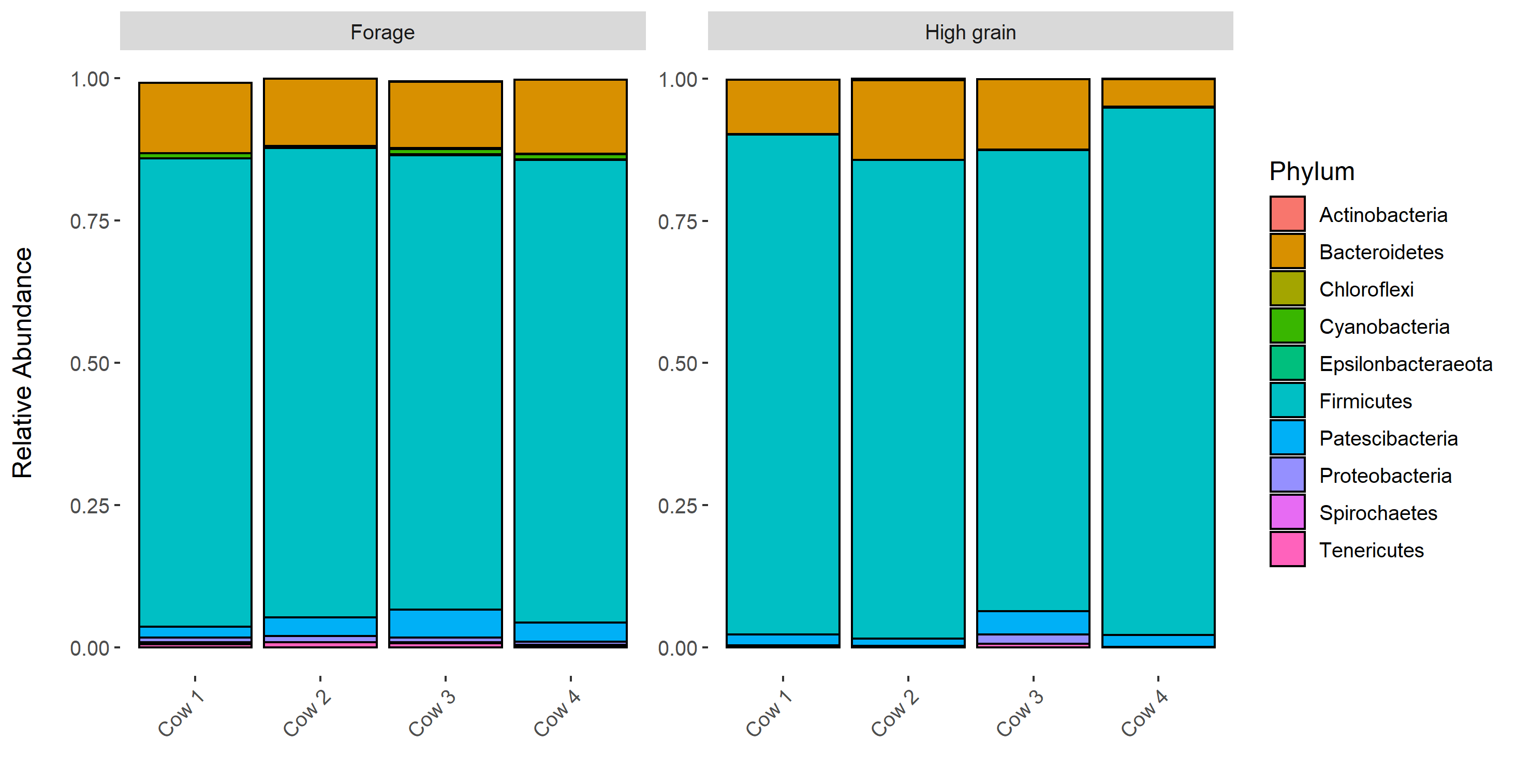


**Supplementary Figure S2.** Heatmap of the correlation matrix between the 21 differentially abundant genera and the miRNAs identified in the rumen fluid. The hierarchical clustering shows two main clusters.


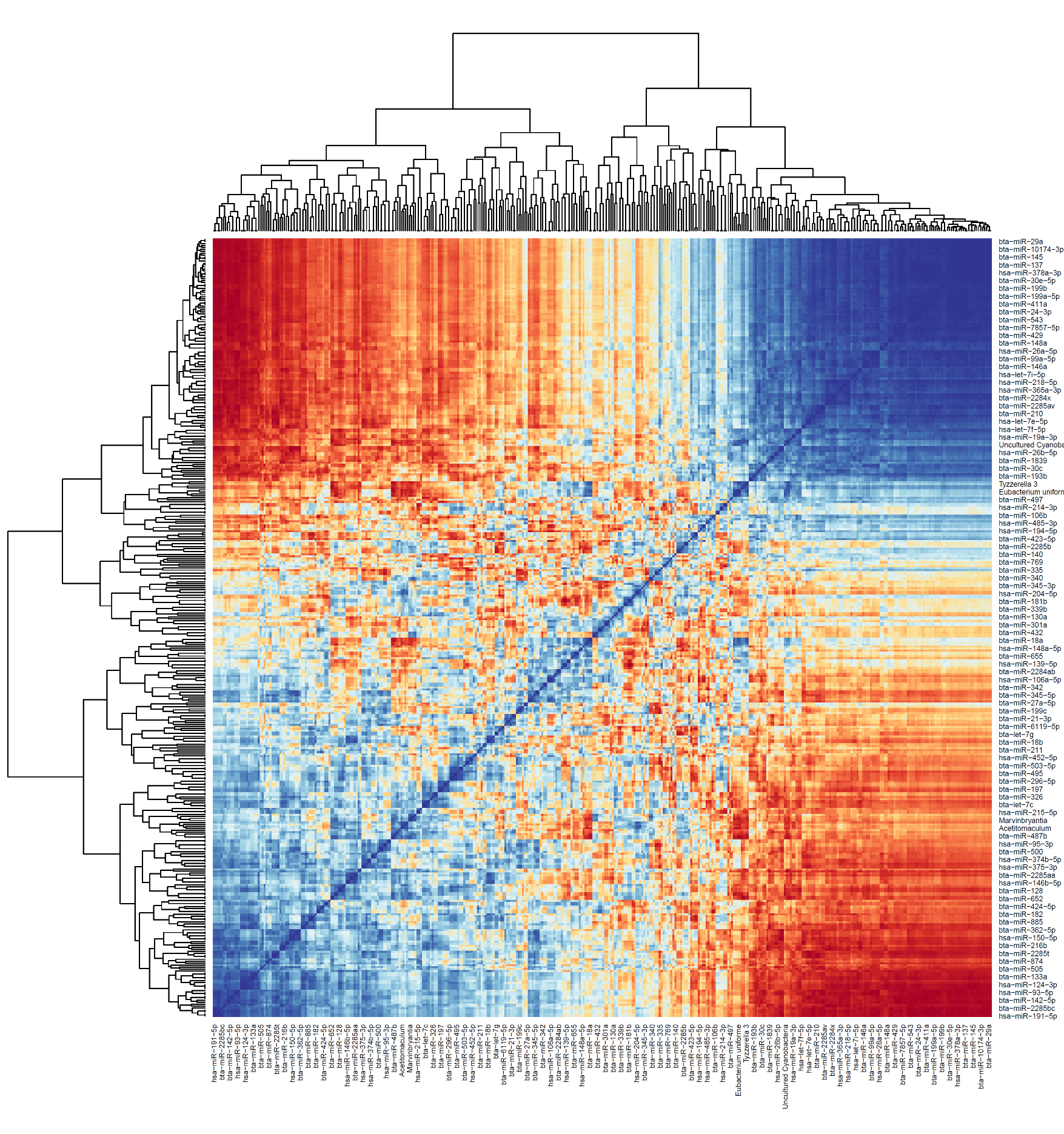


**Supplementary Figure S3.** Correlation network showing the significant (P < 0.05) and strongly positive (r > 0.70) correlations between miRNAs normalized read counts and the relative abundances of the 30 most abundant microbial predicted pathways and of the 4 pathways that were found in common between the
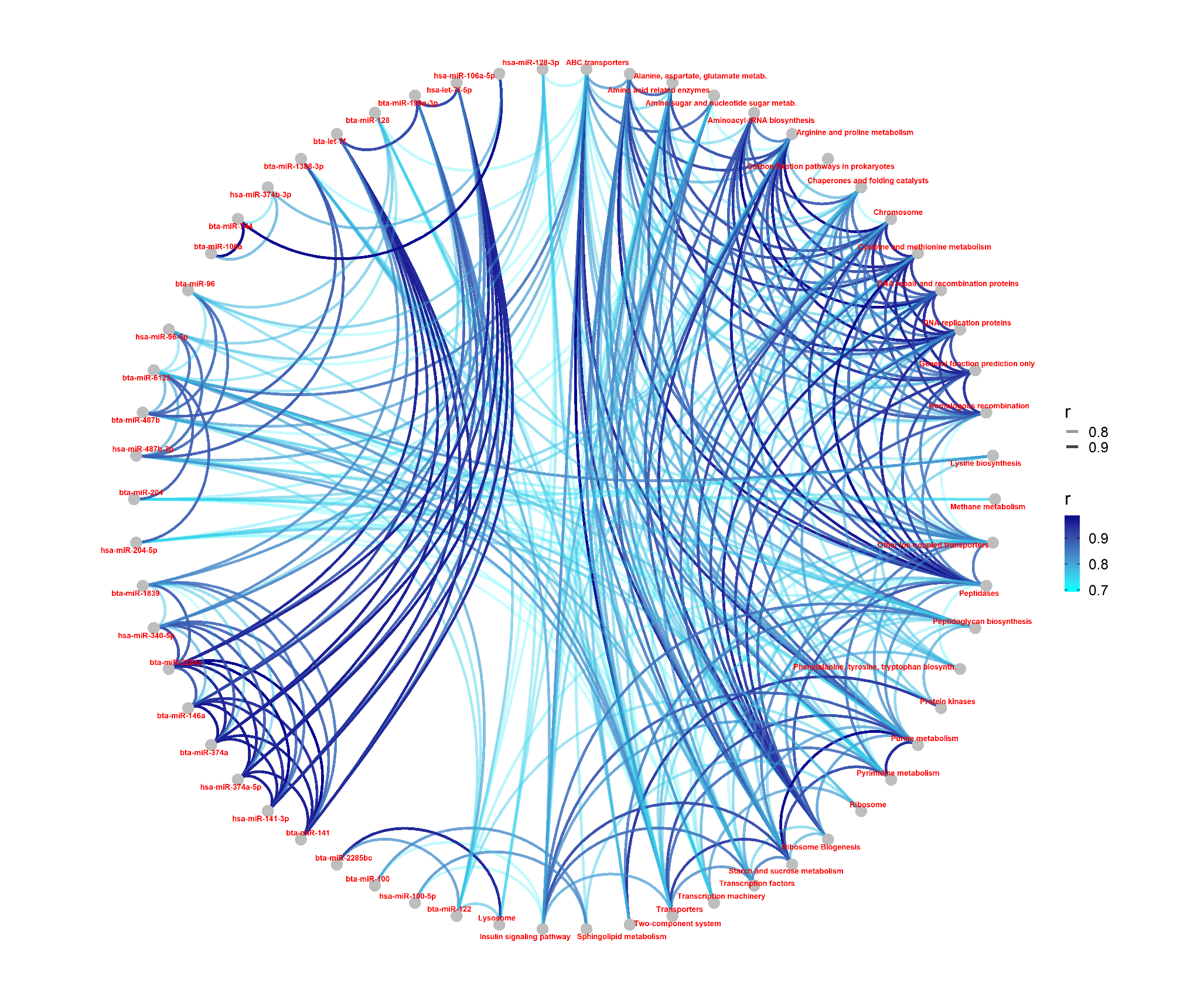
miRNAs functional prediction and CowPI predictions.
